# Solar thermal fuels: azobenzene as a cyclic photon–heat transduction platform

**DOI:** 10.3762/bjoc.21.159

**Published:** 2025-10-08

**Authors:** Jie Yan, Shaodong Sun, Minghao Wang, Si Wu

**Affiliations:** 1 Hefei National Research Center for Physical Sciences at the Microscale, Anhui Key Laboratory of Optoelectronic Science and Technology, Department of Polymer Science and Engineering, University of Science and Technology of China, Hefei 230026, China; 2 Research Center on Advanced Chemical Engineering and Energy Materials, China University of Petroleum (East China), 266580, Qingdao, China; 3 School of Public Security and Emergency Management, Anhui University of Science and Technology, 231131, Hefei, China

**Keywords:** azobenzene, energy, photoresponsive, solar thermal fuels

## Abstract

Azobenzene-based solar thermal fuels have undergone significant advancements over the past four decades, emerging as a promising technology for light-to-thermal energy conversion. While these materials exhibit considerable development potential, critical challenges remain that hinder their practical implementation. In this perspective, we systematically analyze four representative azobenzene-based solar thermal fuel systems including nanocarbon-hybrid, conjugated polymer, linear polymer, and small-molecule derivative formulations to trace their developmental trajectories and identify key limitations. Through this comparative analysis, we aim to clarify the current state of azobenzene-based solar thermal fuels, while mapping strategic pathways for future technological advancements in this rapidly evolving research field.

## Introduction

Solar energy occupies a pivotal position among renewable energy sources. To date, solar energy conversion and storage technologies have experienced considerable progress, including artificial photosynthesis [1], solar thermal collectors [2], photovoltaic cells [3], and solar thermal fuels [4–5]. Among these innovations, solar thermal fuels based on photoswitchable molecules are highly promising candidates for capturing and storing solar energy as heat [6–7].

**Figure 1:**
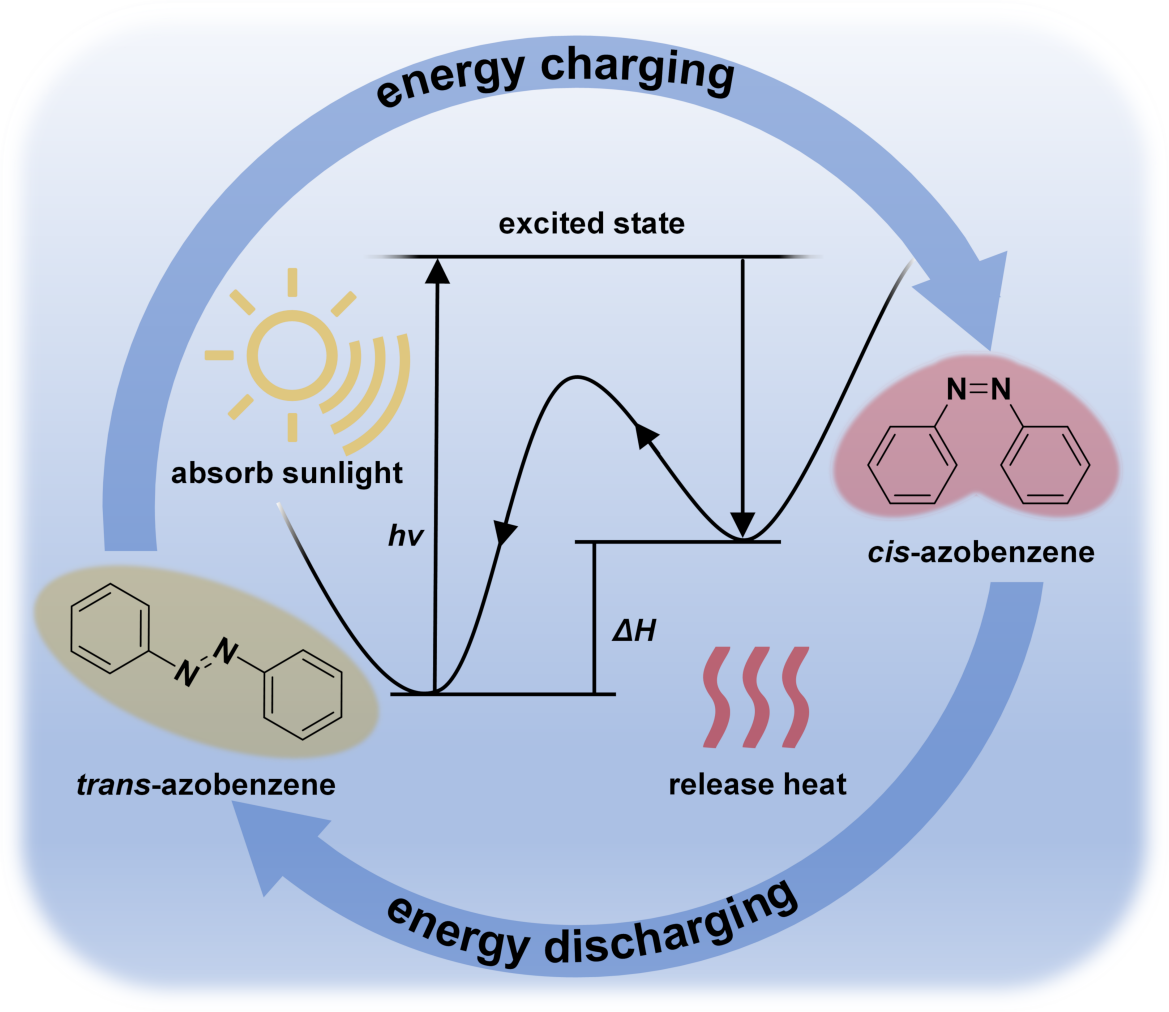
Schematic diagram of molecular solar thermal energy storage system.

In such systems, low-energy ground-state molecules absorb solar radiation and undergo isomerization to form high-energy photoexcited states (metastable configurations) through structural or bond rearrangements, thereby enabling solar energy storage. This reversible process is termed "energy charging", while the subsequent transformation of high-energy photoisomers back to their ground state – triggered by external stimuli such as light, heat, or catalysts – releases stored thermal energy in a controlled manner, a phase designated "energy discharging" (Figure 1). The core advantages of such closed-loop systems are primarily manifested in their reversible cycling capability, carbon neutrality characteristics, transportation convenience, and on-demand heat generation [8].

**Figure 2:**
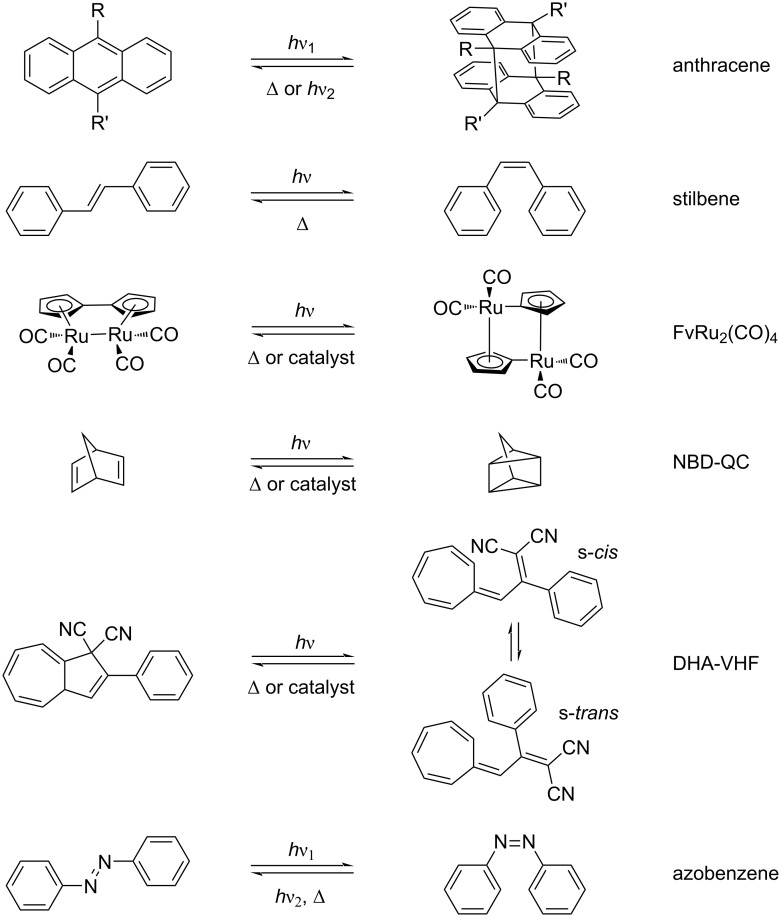
Photoisomerization of different types of molecular optical switches. Figure 2 was redrawn from [8].

Significant progress has been made in developing molecular photochromic systems for solar thermal energy storage, with representative examples including anthracene derivatives [9–11], diphenylethylene [12–13], fulvalene-based tetracarbonyldiruthenium (FvRu_2_(CO)_4_) [14–16], norbornadiene–quadricyclane (NBD–QC) complexes [17–23], dihydroazulene–vinylheptadiene (DHA-VHF) systems [24–30], and azobenzene derivatives [31–34] (Figure 2). In addition, other reversible photoisomers exhibit potential for development as solar thermal fuels [35–41]. Notably, the utilization of azobenzene-based materials in solar thermal storage applications is gaining considerable attention due to their advantages in low synthesis cost, facile molecular design, exceptional cycling stability, and superior fatigue resistance [6,8].

Since Olmsted’s pioneering exploration of azobenzene compounds as solar thermal fuels in 1983 [42], significant breakthroughs have been achieved through interdisciplinary integration of organic synthesis, functional materials engineering, photophysical mechanism analysis, and computational chemistry. So far, azobenzene-based solar thermal fuels mainly include: nanocarbon-based azobenzene polymers, conjugated azobenzene polymers, linear azobenzene polymers, and azobenzene small molecule derivatives. From this perspective, we review various azobenzene-based solar thermal fuels and their potential applications, highlighting key contributions in the field.

## Discussion

### Nanocarbon-based azobenzene polymer solar thermal fuels

**Figure 3:**
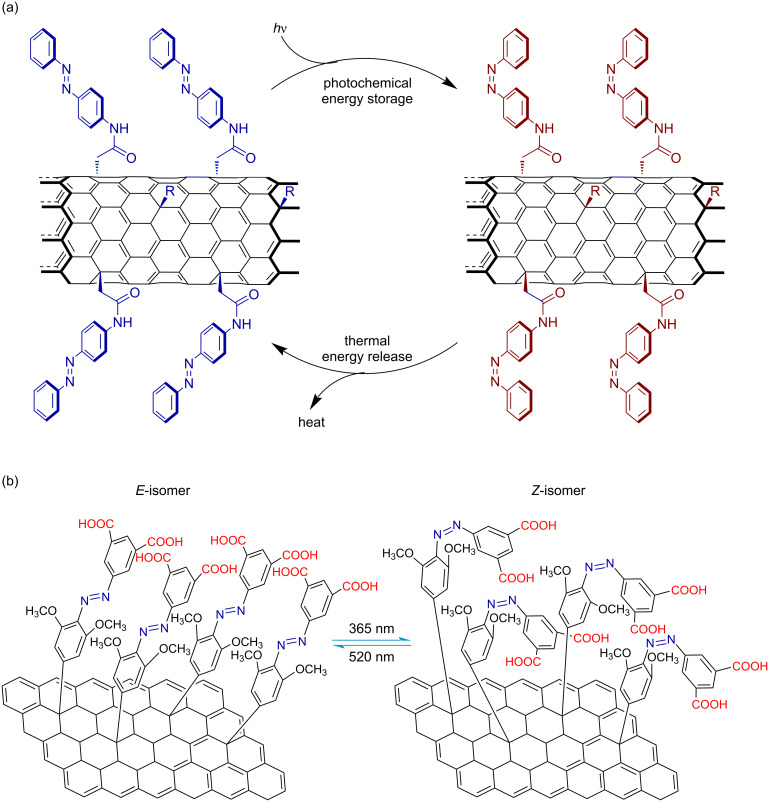
Nanocarbon-based azobenzene polymer solar thermal fuels: (a) SWCNT templating. Figure 3a is from [43] (T. J. Kucharski et al., “Templated assembly of photoswitches significantly increases the energy-storage capacity of solar thermal fuels“, *Nature Chemistry*, vol. 6, pages 441–447, published by Springer Nature, 2014, reproduced with permission from SNCSC). This content is not subject to CC BY 4.0. (b) RGO templating. Figure 3b was used with permission of The Royal Society of Chemistry, from [46] (“High-energy, stable and recycled molecular solar thermal storage materials using AZO/graphene hybrids by optimizing hydrogen bonds” by W. Luo et al., *Nanoscale*, vol. 7, © 2015); permission conveyed through Copyright Clearance Center, Inc. This content is not subject to CC BY 4.0.

Nanocarbon-based azobenzene polymer solar thermal fuels are constructed by grafting azobenzene moieties onto carbon nanotube [43–45] or reduced graphene oxide (RGO) templates. Grossman et al. synthesized azobenzene-functionalized single-walled carbon nanotubes (SWCNTs) (Figure 3a) and demonstrated their application as solar thermal fuels [43]. The conformational restriction imposed by the chromophore-template structure combined with photochemically generated spatial strain enables each azobenzene unit to store >200% more energy than isolated molecules, while achieving multi-order-of-magnitude improved storage lifetime and resistance to material degradation during repeated cycling. Feng and co-workers covalently grafted azobenzene groups onto RGO templates (Figure 3b) and investigated their solar thermal energy storage performance [46]. Key parameters governing the performance of azobenzene–RGO hybrid solar thermal fuels include hydrogen-bonding interactions, intermolecular distances between adjacent azobenzene groups, and electronic coupling effects.

### Conjugated azobenzene polymer solar thermal fuels

**Figure 4:**
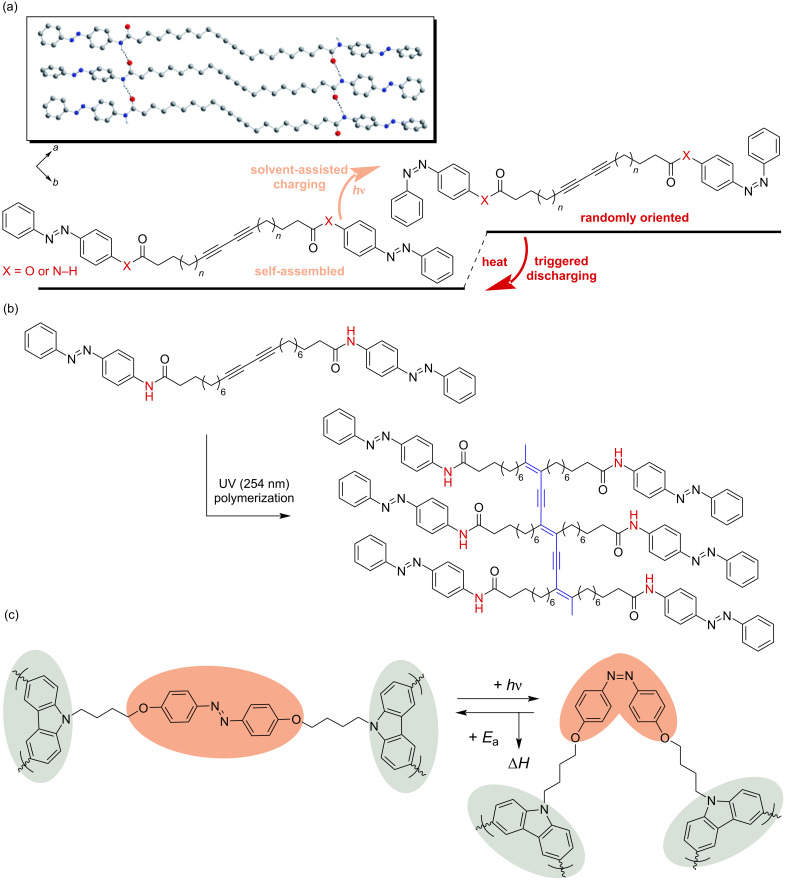
Conjugated azobenzene polymer solar thermal fuels: (a) Photoisomerization and thermally induced reverse isomerization of diacetylene monomers. The inset depicts the crystal structure of diacetylene monomer (*n* = 6, X = N–H) with dashed lines indicating intermolecular hydrogen bonds. Figure 4a was used with permission of The Royal Society of Chemistry, from [47] (“Photon energy storage materials with high energy densities based on diacetylene–azobenzene derivatives” by G. D. Han et al., *Journal of Materials Chemistry A*, vol. 4, © 2016); permission conveyed through Copyright Clearance Center, Inc. This content is not subject to CC BY 4.0. (b) Schematic illustration of diacetylene monomer photopolymerization and the conjugated polydiacetylene structure. Figure 4b was redrawn from [47]. (c) Photoisomerization of a conjugated azobenzene polymer with a carbazole backbone. Figure 4c was redrawn from [48].

Conjugated azobenzene polymer solar thermal fuels [47–48] utilize π-conjugated systems as templates for azobenzene chromophore photoisomerization, as schematically illustrated in Figure 4. Han et al. developed azobenzene-functionalized polydiacetylene-based solar thermal fuels [47], where the azobenzene moieties undergo reversible photoisomerization and thermally induced reverse isomerization, enabling "energy charging–discharging" cycles (Figure 4a). Under ultraviolet (UV) irradiation (254 nm), the closely packed diacetylene polymers undergo polymerization to form π-conjugated polydiacetylene structures, with an energy storage density reaching 243 J/g (Figure 4b). These azobenzene-functionalized polydiacetylenes exhibit structural analogy to azobenzene-modified carbon nanotubes and graphene in terms of photoresponsive behavior. Sert et al. combined electroactive and light-harvesting carbazole units with photoresponsive azobenzene units in a unique macromolecular architecture (Figure 4c) [48]. The resulting cross-linked polycarbazole structure led to a high solar thermal storage capacity of 179.9 J/g and an extended half-life at 60 °C, increasing from 7 min for the monomer to 103 min for the polymer.

### Linear azobenzene polymer solar thermal fuels

**Figure 5:**
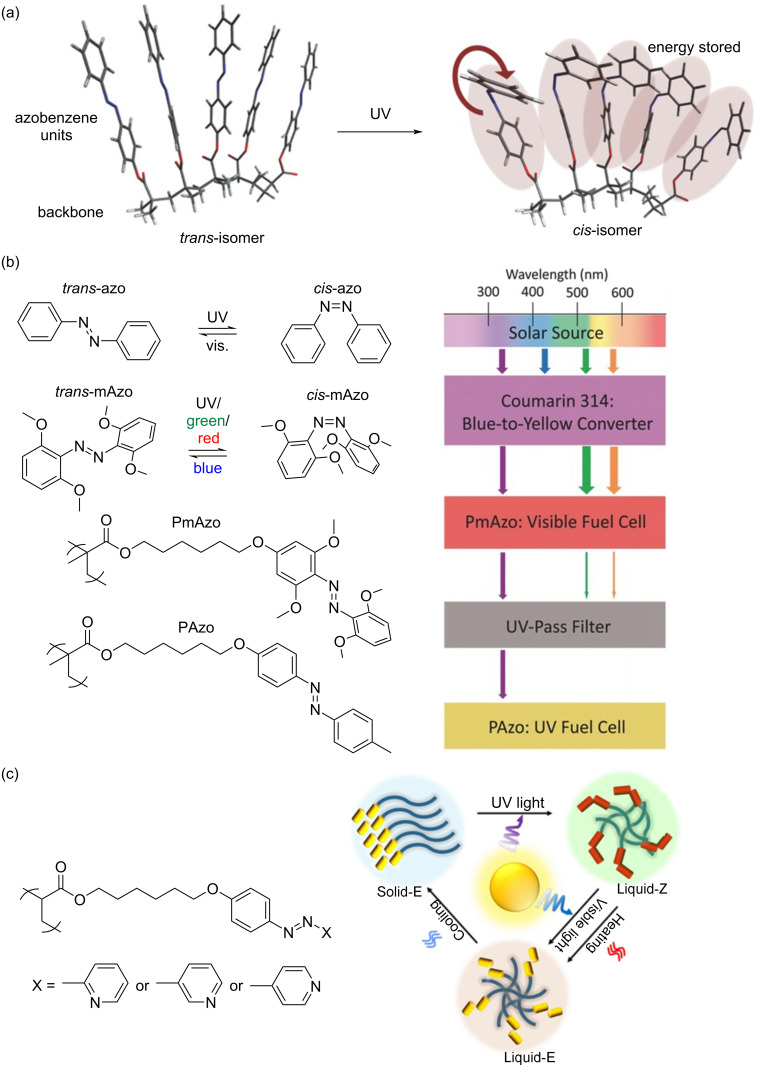
Linear azobenzene polymer solar thermal fuels: (a) Schematic illustration of the *trans*-to-*cis* isomerization of an azobenzene polymer upon UV illumination. Figure 5a was reproduced from [58], D. Zhitomirsky et al., “Solid-State Solar Thermal Fuels for Heat Release Applications”, *Adv. Energy Mater.*, with permission from John Wiley and Sons. Copyright © 2015 WILEY-VCH Verlag GmbH & Co. KGaA, Weinheim. This content is not subject to CC BY 4.0. (b) Structure of the UV–vis solar-thermal cell. Figure 5b was adapted from [59], A. K. Saydjari et al., “Spanning the Solar Spectrum: Azopolymer Solar Thermal Fuels for Simultaneous UV and Visible Light Storage”, *Adv. Energy Mater.*, with permission from John Wiley and Sons. Copyright © 2016 WILEY-VCH Verlag GmbH & Co. KGaA, Weinheim. This content is not subject to CC BY 4.0. (c) Schematic of the mechanism for photothermal energy and phase change energy storage of azopyridine polymers. Figure 5c was adapted from [60], R. Liang et al., “Azopyridine Polymers in Organic Phase Change Materials for High Energy Density Photothermal Storage and Controlled Release”, *Angew. Chem., Int. Ed.*, with permission from John Wiley and Sons. Copyright © 2024 Wiley-VCH GmbH. This content is not subject to CC BY 4.0.

Linear azobenzene polymers possess excellent processability and serve as ideal solid-state matrices for photoisomerization [49–57], making them suitable candidates for solar thermal fuels. Zhitomirsky et al. studied a linear azobenzene polymer-based solid solar thermal fuel [58], which enables the fabrication of uniform thin films capable of storing up to 108 J/g of thermal energy (Figure 5a). Wu et al. designed an azobenzene polymer-based device capable of efficiently storing both UV and visible light under sunlight irradiation [59], achieving a solar efficiency of 0.4% and a solid-state isomerization efficiency of 72.7% (Figure 5b). Feng and co-workers reported the synthesis of three azopyridine polymers (*ortho*-, *meta*-, and *para*-) [60], with the *m*-azopyridine polymer exhibiting a significant photothermal energy storage capacity of 430 J/g (Figure 5c). This study presents potential implications for energy distribution and utilization in domestic consumer applications, and for photothermal-assisted insulation strategies.

### Azobenzene small molecule derivative solar thermal fuels

Azobenzene small-molecule derivatives can be systematically categorized based on molecular structure into monoazobenzene derivatives [61–69], multiazobenzene derivatives [70–73], heteroaromatic azobenzene derivatives [74–81], and macrocyclic azobenzene derivatives [82–88]. From an energy charging perspective, some solvent-free systems store energy in the solid state [89] or via photoinduced solid–liquid phase transitions [61,69,74], which occur because the melting temperature (*T_m_*) of *trans*-azobenzene is above room temperature and the *T_m_* of the isomerized *cis*-azobenzene is below room temperature, while others require solvent-assisted charging mechanisms [70–71]. This structural and functional diversity makes them particularly valuable for solar thermal storage applications.

**Figure 6:**
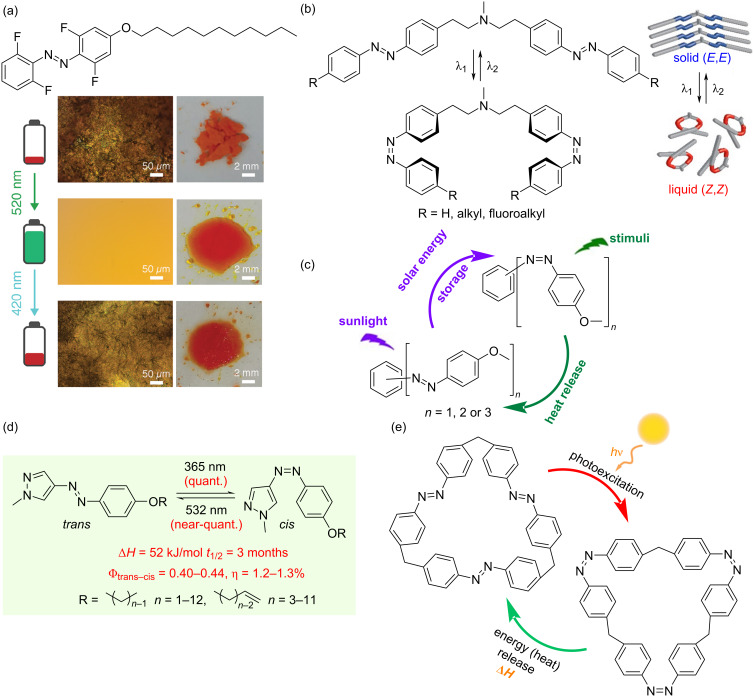
Representative examples of azobenzene small-molecule derivative solar thermal fuels. (a) Polarized light microscopy images and photographs of the solid–liquid transition of fluorinated azobenzene under visible light. Figure 6a was adapted from [65], Y. Wu et al., “An Innovative Azobenzene‐Based Photothermal Fabric with Excellent Heat Release Performance for Wearable Thermal Management Device”, *Small*, with permission from John Wiley and Sons. Copyright © 2024 Wiley-VCH GmbH. This content is not subject to CC BY 4.0. (b) Schematic illustration of the isomerization of the bisazobenzene small molecule. Figure 6b was reproduced from [90] (© 2023 A. Gonzalez et al., published by American Chemical Society, distributed under the terms of the Creative Commons Attribution-NonCommercial-NoDerivatives 4.0 International license, https://creativecommons.org/licenses/by-nc-nd/4.0/). This content is not subject to CC BY 4.0. (c) Photoisomerization and thermal reverse isomerization of small molecules incorporating diverse azo units. Figure 6c was used with permission of The Royal Society of Chemistry, from [91] (“Photoswitches with different numbers of azo chromophores for molecular solar thermal storage” by S. Sun et al., *Soft Matter*, vol. 18, © 2022); permission conveyed through Copyright Clearance Center, Inc. This content is not subject to CC BY 4.0. (d) Arylazopyrazole-based azobenzene derivatives. Figure 6d was adapted with permission from [74] Copyright © 2020 American Chemical Society. This content is not subject to CC BY 4.0. (e) Molecular structure of the trisazobenzenophane. Figure 6e was adapted with permission from [82] Copyright © 2013 American Chemical Society. This content is not subject to CC BY 4.0.

Feng et al. incorporated fluorinated azobenzene small molecules into polymer fibers [65], enabling solvent-free charging and discharging under visible light (Figure 6a). This material exhibited good capacity for releasing high-temperature heat (80–95 °C) at room temperature and in cold environments, along with an energy storage lifetime approaching two years, significantly exceeding the reported storage lifetime of other azobenzene-based solar thermal fuels. Han et al. synthesized a series of bisazobenzene small molecules with various substituents [90], all exhibiting photoinduced solid–liquid phase transitions. In contrast, comparable monoazobenzene small molecules showed substituent-dependent photoinduced solid–liquid phase transition behavior. This provides a feasible strategy for enhancing the energy density of azobenzene-based solar thermal fuels by controlling phase transitions (Figure 6b). Wu et al. conducted a comparative study on monoazobenzene versus multiazobenzene derivatives [91], demonstrating that in solution-assisted conditions, bisazobenzene derivatives achieve a peak energy density of 272 J/g. However, in the solid state, energy storage is limited to monoazobenzene derivatives due to photoinduced solid–liquid transition (Figure 6c), providing new insights into the structure–property relationships in azobenzene systems. Li et al. reported a series of rationally designed arylazopyrazole-based azobenzene derivatives [74] that enable simultaneous photon and environmental heat capture through their photoinduced solid–liquid transition behavior, achieving high gravimetric energy densities (300–400 J/g) with long-term storage stability (Figure 6d). Grossman and Durgun proposed methods for incorporating azobenzenes into macrocyclic structures [82]. Their computational models indicated that the molecular rings connecting the molecules can improve energy density by imposing strain within the molecule. However, there are no experimental reports of these compounds and their synthesis is expected to remain a challenge in the future.

### Applications of azobenzene-based solar thermal fuels

**Figure 7:**
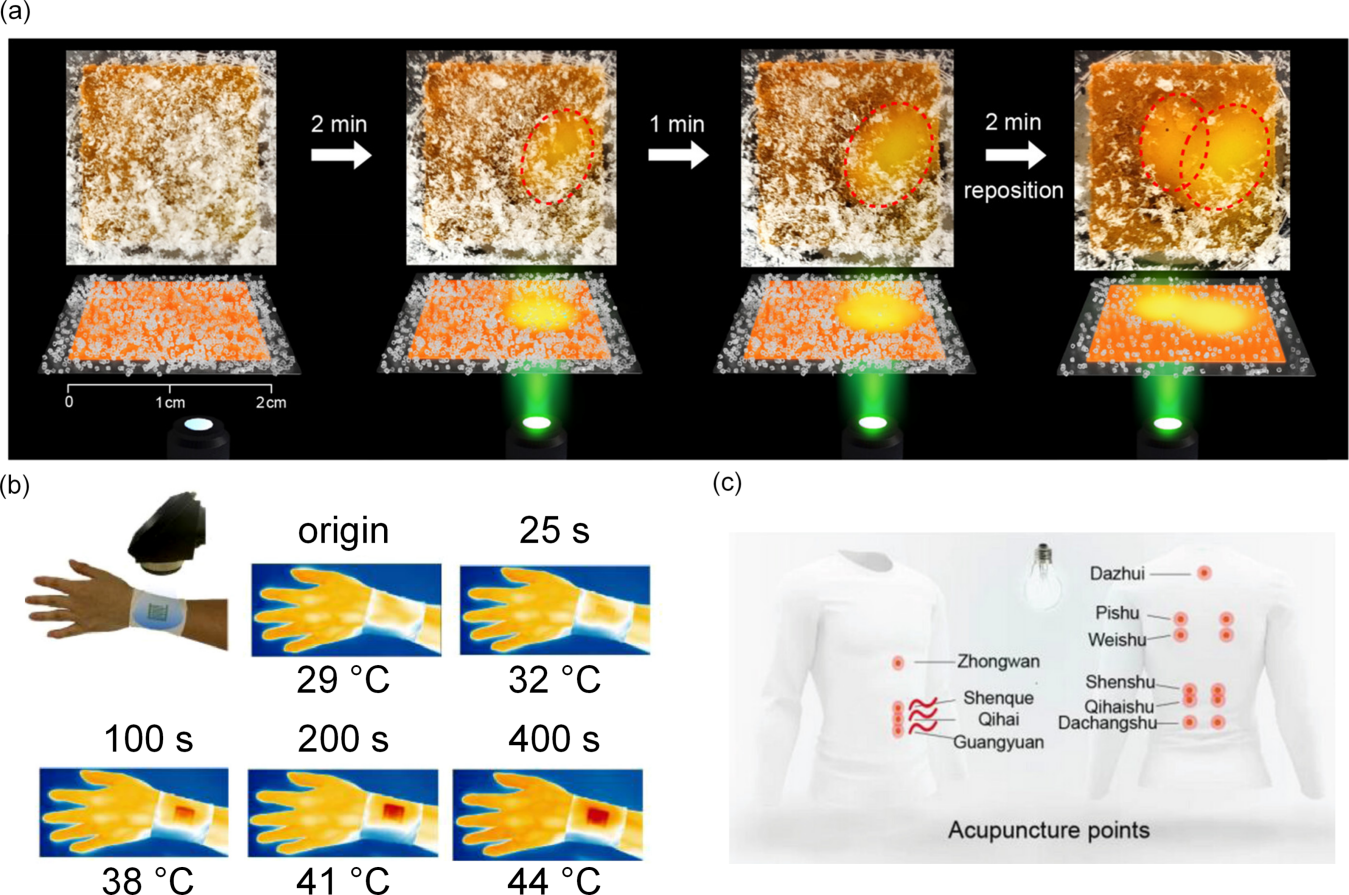
(a) Deicing test of charged solar thermal fuels under green light irradiation (550 nm). Figure 7a was reprinted with permission from [74] Copyright © 2020 American Chemical Society. This content is not subject to CC BY 4.0. (b) Real-time infrared thermal imaging images of a charged self-heating wristband under blue light irradiation (450 nm). Figure 7b was reprinted from [94], *Energy Storage Materials*, vol. 42, by L. Fei, Y. Yin, M. Yang, S. Zhang, C. Wang, “Wearable solar energy management based on visible solar thermal energy storage for full solar spectrum utilization“, p. 636–644, Copyright (2021), with permission from Elsevier. This content is not subject to CC BY 4.0. (c) Schematic of the fabric with the printed acupuncture point pattern. Figure 7c was reproduced from [95], L. Fei et al., “Efficient and Robust Molecular Solar Thermal Fabric for Personal Thermal Management”, *Advanced Materials*, with permission from John Wiley and Sons. Copyright © 2023 Wiley-VCH GmbH. This content is not subject to CC BY 4.0.

After more than four decades of research, our understanding of azobenzene-based solar thermal fuels has significantly advanced, driving continuous innovation in this field. The application scope of these materials has expanded beyond initial temperature monitoring [58,92–93] to practical energy utilization scenarios. For instance, Li et al. utilized charged solar thermal fuels for deicing tests [74], showing effective ice removal capability under green light irradiation (Figure 7a). Wang et al. developed a photostimulated self-heating wristband [94] that elevates surface temperature from 29 °C to 44 °C upon blue light irradiation, protecting users from frostbite (Figure 7b); and Li and Wang et al. fabricated fabrics containing azobenzene-based solar thermal fuels that exhibit high energy storage density and long half-lives [95], enabling photothermal stimulation for moxibustion at acupoints upon light irradiation (Figure 7c). These examples collectively highlight the transition from fundamental studies to real-world applications, where azobenzene-based systems now serve as enabling technologies for device deicing, wearable thermal management, and wearable smart textiles for acupuncture.

## Conclusion and Future Directions

We summarized the development of azobenzene-based solar thermal fuels and demonstrated their potential applications. Nanocarbon-integrated azobenzene polymers: Their energy storage efficiency (up to >200%) and cycle life are primarily modulated by template confinement effects and electronic coupling. However, high costs and challenges in large-scale fabrication are inherent limitations. Conjugated azobenzene polymers undergo photoisomerization and thermally triggered back-isomerization, enabling cyclic "charging–discharging" of energy. However, their solid-state phase transition behavior is inherently governed by molecular packing, making solvent-independent energy cycling difficult to achieve. Linear azobenzene polymers exhibit excellent processability and solid-state photoisomerization performance. However, their moderate energy density is inherently limited by the mass fraction of non-active polymer segments. Small-molecule azobenzene derivatives achieve gravimetric energy densities of 300–400 J/g with long-term storage stability. However, solvent dependence or steric hindrance in multiazobenzene units remains an inherent challenge dictated by material type. These materials show promising applications in device deicing, wearable thermal management, and wearable smart textiles for acupuncture.

While azobenzene-based solar thermal fuels excel in molecular designability and cycling stability, they exhibit significant gaps in spectral response range and energy density compared to non-azobenzene systems. These comparisons highlight future directions for cross-material integration. The reversible *trans*–*cis* isomerization of azobenzene makes it an ideal model system for studying solar thermal fuels, as it enables both solar energy storage and heat release. While significant advances have been made, critical challenges persist. First, a major limitation is that most azobenzene-based solar thermal fuels can only store UV or visible light, which represent a minor portion of the solar spectrum. In contrast, approximately 50% of the solar irradiance is in the form of near-infrared (NIR) radiation, highlighting the urgent need for NIR-responsive systems. Second, although azobenzene-based fuels have evolved from conceptual designs to functional materials, many still require solvent assistance during energy charging. The use of organic solvents exacerbates environmental concerns, increases costs, complicates operation, and hinders practical deployment. Third, azobenzene groups release energy through a process involving thermal relaxation or thermally induced *cis*–*trans* isomerization. Temperature critically governs the efficiency and kinetics of this process: elevated temperatures accelerate uncontrolled instantaneous heat release, while low temperatures induce phase transition-induced failures that compromise structural stability. Despite tunable photothermal parameters, achieving controlled heat release and structural robustness under extreme temperatures remains a pivotal challenge for azobenzene systems. Looking forward, improving the energy storage density of azobenzene-based solar thermal fuels remains an important research direction. However, significant opportunities exist for developing azobenzene derivatives that absorb visible light to NIR. In terms of charging and discharging methods, solvent-free systems capable of efficient charging and discharging in the solid state or via photoinduced solid–liquid transitions will be advantageous. Simultaneously, enhancing the long-term stability of azobenzene energy storage is a key area for future research.

## Data Availability

Data sharing is not applicable as no new data was generated or analyzed in this study.
